# Somatostatin Receptor SSTR-2a Expression Is a Stronger Predictor for Survival Than Ki-67 in Pancreatic Neuroendocrine Tumors

**DOI:** 10.1097/MD.0000000000001281

**Published:** 2015-10-09

**Authors:** Shreya Mehta, Philip R. de Reuver, Preetjote Gill, Juliana Andrici, Lisa D’Urso, Anubhav Mittal, Nick Pavlakis, Stephen Clarke, Jaswinder S. Samra, Anthony J. Gill

**Affiliations:** From Department of Gastrointestinal Surgery, Royal North Shore Hospital and North Shore Private Hospital, University of Sydney, New South Wales, Australia (SM, PDR, PG, AM, JSS); Department of Medical Oncology, Royal North Shore Hospital and North Shore Private Hospital, University of Sydney, New South Wales, Australia (NP, SC); Macquarie University Hospital, Macquarie University, New South Wales, Australia (JSS); and Department of Anatomical Pathology Royal North Shore Hospital, Cancer Diagnosis and Pathology Group, Kolling Institute of Medical Research, University of Sydney, New South Wales, Australia (JA, LDU, AJG)

## Abstract

Somatostatin receptors (SSTR) are commonly expressed by neuroendocrine tumors. Expression of SSTR-2a and SSTR-5 may impact symptomatic management; however, the impact on survival is unclear. The aim of this study is to correlate SSTR-2a and SSTR-5 expression in pancreatic neuroendocrine tumors (PNETs) with survival.

This study is designed to determine the prognostic significance of somatostatin receptors SSTR-2a and SSTR-5 in PNETs.

This retrospective cohort study included cases of resected PNETs between 1992 and 2014. Clinical data, histopathology, expression of SSTR and Ki-67 by immunohistochemistry, and long-term survival were analyzed.

A total of 99 cases were included in this study. The mean age was 57.8 years (18–87 years) and median tumor size was 25 mm (range 8–160 mm). SSTR-2a and SSTR-5 expression was scored as negative (n = 19, 19.2%; n = 75, 75.8%, respectively) and positive (n = 80, 80.1%; n = 24, 24.2%). The median follow-up was 49 months. SSTR-2a expression was associated with improved overall survival, with cumulative survival rates at 1, 3, and 5 years being 97.5%, 91.5%, and 82.9%, respectively. Univariate analysis demonstrated better survival in SSTR-2a positive patients (log rank *P* = 0.04). SSTR-5 expression was not associated with survival outcomes (log rank *P* = 0.94). Multivariate analysis showed that positive SSTR-2a expression is a stronger prognostic indicator for overall survival [Hazard Ratio (HR): 0.2, 95% Confidence interval (CI): 0.1–0.8] compared to high Ki-67 (HR: 0.8, 95% CI: 0.1–5.7).

Expression of SSTR-2a is an independent positive prognostic factor for survival in PNETs.

## INTRODUCTION

Pancreatic neuroendocrine tumors (PNETs) are an increasingly recognized clinical problem, yet they remain poorly understood.^1^ PNETs account for 1% to 3% of all pancreatic neoplasms and have one of the lowest 5-year survival rates of all gastroenteropancreatic neuroendocrine tumors, ranging from 42% to 71%, with a worse prognosis associated with an advanced tumor stage and nonfunctioning PNETs.^1,2^ Recognized adverse prognostic indicators include increased tumor size, vascular invasion, distant metastases, and a high Ki-67 labeling index.^3^ However, much is yet to be learned about the underlying pathophysiology involved in the progression and prognosis of PNETs.^4^

Somatostatin is a peptide hormone that inhibits growth and hormone secretion by neuroendocrine cells. Somatostatin analogs such as octreotide and lanreotide are commonly used as therapies to inhibit tumor growth and to provide symptomatic relief in functional PNETs by reducing hormone hypersecretion.^5,6^ The rationale for their use is based on the high levels of somatostatin receptors (SSTR) that have been identified in neuroendocrine tumors.^7^ SSTR-2a and SSTR-5 have received considerably more attention than the other somatostatin receptors, given that currently available somatostatin analogs have a high affinity to SSTR-2a and SSTR-5.^8^ Although it is well established that activation of these receptors by somatostatin analogs improves the hormonal symptoms exerted by functional tumors, there is little data to validate the role that SSTR-2a and SSTR-5 directly play on prognosis and survival outcome.^8,9^

We hypothesize that SSTR-2a and SSTR-5 expression is associated with improved overall survival. The aim of this study is to determine the expression of SSTR-2a and SSTR-5 in PNET and to assess their impact on survival.

## METHODS

### Patients

#### Inclusion Criteria

Patients undergoing surgical resection for PNET between 1992 and 2014 at Royal North Shore Hospital (RNSH) and affiliated institutions that had adequate tissue in formalin-fixed paraffin-embedded (FFPE) tissue blocks were included in the present study.

#### Exclusion Criteria

In cases where patients had more than 1 PNET at the time of surgical resection, the tumor with the highest grade was studied. If there was more than 1 tumor with the same grade present, then the tumor with the stage was included in this study.

#### Data Collection

All PNETs underwent independent pathological review to confirm the pathological diagnosis, grade, and stage. Clinical follow-up was obtained from medical records or the surgeon's private rooms. The following data were extracted on a structure pro forma: number of tumors, tumor size, stage, vascular invasion, peri-neural invasion, extrapancreatic extension, and lymph node involvement. Tumors were graded as per the World Health Organisation (WHO) 2010 classification.^10^ In grade-discrepant tumors, where grade assigned by mitotic count differed from the grade assigned by Ki-67 proliferation index, the tumors were assigned the higher grade (in all cases the Ki-67 proliferation index). Tumors were staged based on the seventh edition 2009 American Joint Committee on Cancer / Union for International Cancer Control Tumor - node - metastasis (TNM) classification.^11^

### Immunohistochemistry

Tissue microarrays (TMAs) were constructed containing two 1 mm cores from each tumor. Immunohistochemistry for Ki-67, SSTR-2a, and SSTR-5 was performed using previously described methods.^12,13^ Briefly, immunochemistry for SSTR2a (Clone UMB-1, Epitomics, Inc., Burlingame CA) and SSTR-5 (Clone UMB-4 Epitomics, Inc.) was performed using commercially available rabbit monoclonal antibodies at dilutions of 1 in 100 after heat-induced epitope retrieval at 97 °C for 30 minutes in acidic retrieval solution Epitope solution (ER)1 (VBS part no. AR9961, Leica Microsystems). For Ki-67 Immunohistochemistry was performed using a mouse monoclonal antibody (clone MIB-1, Dako, CA) after heat-induced epitope retrieval at 97 °C for 30 minutes in the manufacturer's alkaline retrieval solution ER2 (VBS part no. AR9640, Leica Microsystems). The biotin-free Bond Polymer Defined Detection System (DS9713 Leica Microsystems) was used for antigen detection.

SSTR-2a and SSTR-5 immunostaining was scored using a scheme similar to that reported by Korner et al^14^ Briefly, cases were scored as 0 (negative) if no cells demonstrated positive staining, and then semiquantitatively if staining was present from 1+ (weak staining, in <10% of cells) to 2+ (moderate staining, eg, positive at low power but not circumferential at high power) to 3+ (moderate diffuse and strong staining including circumferential staining) to 4+ (intense diffuse and strong staining including circumferential staining). For the purposes of binary analysis, cases that scored 0 or 1 were considered negative, while >1 were considered positive for somatostatin receptor expression. This is based on data using the Korner et al system where an SSTR-2a expression score of 0 or 1 was strongly correlated with negative in vitro ^125^I-[Tyr^3^]-octreotide autoradiography (one gold standard for somatostatin receptor expression) and therefore strongly justifies a negative score whereas scores of 2 or more were considered positive and correlated strongly with positive autoradiography.^14^ For Ki-67, a standard proliferative index (percentage of neoplastic cells which stained positively) was derived.

### Statistical Analysis

The primary endpoint of this study was overall survival, defined as the time from the date of operation to the date of death, or 01/03/2015 irrespective of cause. Kaplan–Meier estimates of survival were obtained. Overall survival was compared between groups with different receptor expression, using log-rank test statistics. Independent prognostic factors for survival were identified using Cox regression analysis including prognostic factors with a *P*-value of <0.1 in the log-rank test. Patient characteristics, histopathological characteristics, and expression of SSTR-2a, SSTR-5, and Ki-67 were factors analyzed to determine effect on the primary endpoint.

*P* values less than 0.05 were considered to indicate statistically significant effects. All statistical analyses were performed using SPSS Version 21.0 (Statistical Package for the Social Sciences, Chicago, IL).

## RESULTS

### Patient Characteristics

The present study included 99 patients, of which 53 were men (53.5%). The mean age was 57.8 years (age range: 18–87 years). Thirty-nine (39.4%) of these patients underwent a pancreaticoduodenectomy (PD), 40 patient (40.4%) had a partial pancreatectomy, 5 patients (5.1%) had a total pancreatectomy, 11 patients (11.1%) had enucleation, and 4 patients (4.0%) had extended resections including pancreatectomies with adrenalectomies (n = 1), partial liver resections (n = 2), and total gastrectomies (n = 1) as an adjunct due to the presence of other pathology in the resected areas. Tumor sites were as follows: 41 (41.4%) in the head of the pancreas, 12 (12.1%) in the neck and/or body of the pancreas, 46 (46.5%) in the tail of the pancreas.

### Tumor Characteristics

The tumor characteristics are summarized in Table 1. The median tumor dimension was 25.5 mm (ranging from 8 mm to 160 mm). Among the 99 tumors, 29 (29.3%) had associated vascular invasion, 6 (6.1%) had perineural invasion and 42 (42.4%) had extrapancreatic extension. Locally resected lymph nodes were positive in 25 patients (25.3%), with a mean lymph node harvest of 7.6 (range 0–37 nodes). There were a total of 15 R1 resections (15.2%). The majority of the tumors were Stage III (33.3%), followed by an equal number of Stage I and Stage II PNETs (30.3%). Most of the PNETs were grade 1 (67.7%), followed by grade 2 (28.3%) then grade 3 (4.0%).

**TABLE 1:**
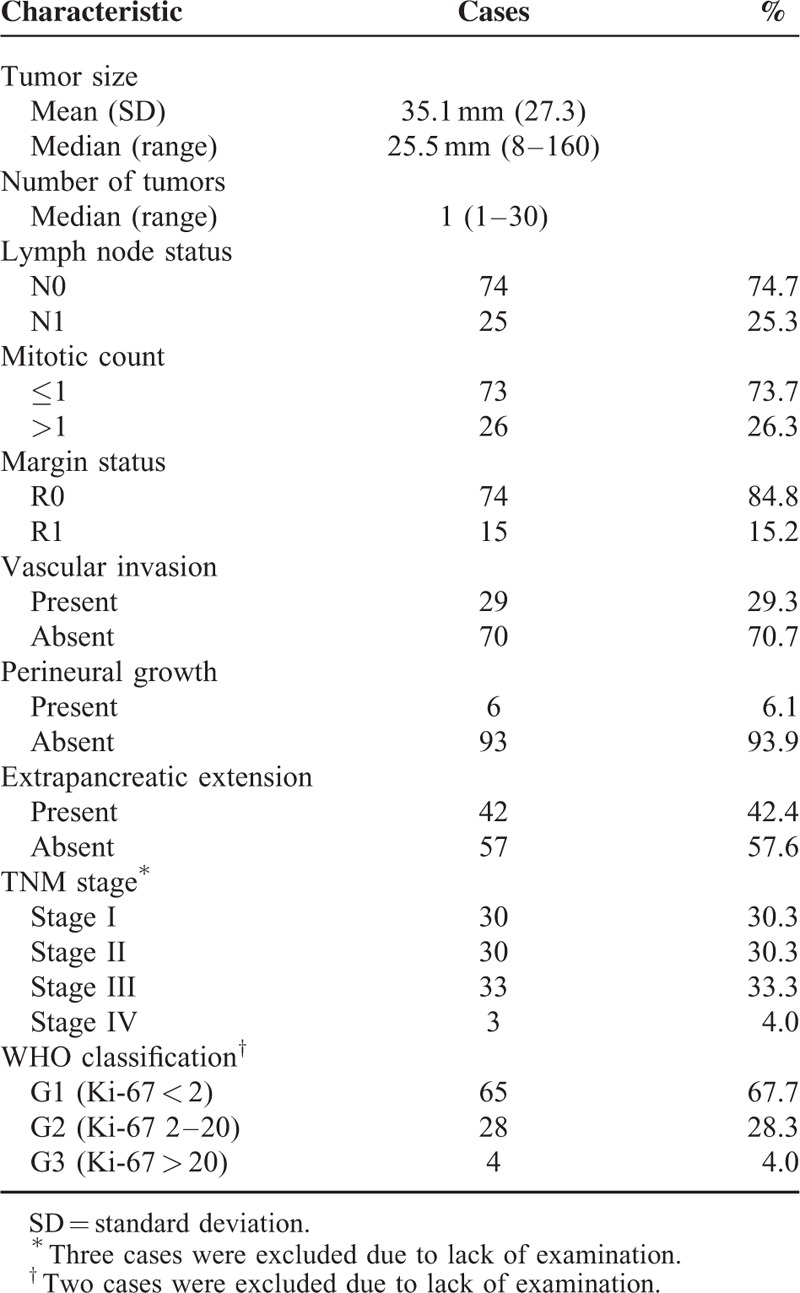
Tumor Characteristics

### Receptor Status

SSTR-2a score was 0 in 14 tumors (14.1%), 1 in 5 tumors (5.1%), 2 in 16 tumors (16.2%), and 3 in 64 tumors (64.6%). SSTR-5 score was 0 in 64 tumors (64.6%), 1 in 11 tumors (11.1%), 2 in 13 tumors (13.1%), and 3 in 11 tumors (11.1%). Table 2 displays the negative and positive receptor status among the tumors, categorized as ≤1 and >1, respectively. Among the tumors classified as grade 3 (n = 4), only 1 case had positive SSTR-2a expression, and none had SSTR-5 expression.

**TABLE 2:**
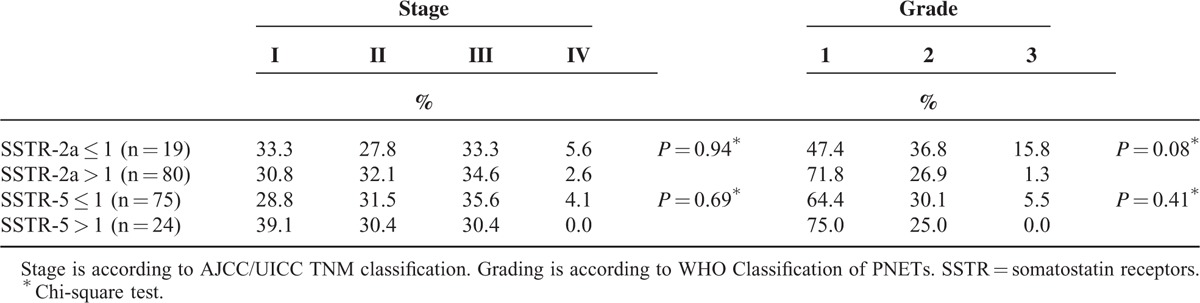
SSTR Expression in PNETs

Further analysis of insulin-producing PNETs and their expression of SSTR-2a and SSTR-5 was performed, but no statistically significant correlation was found between receptor expression and this PNET subtype (Table 3).

**TABLE 3:**
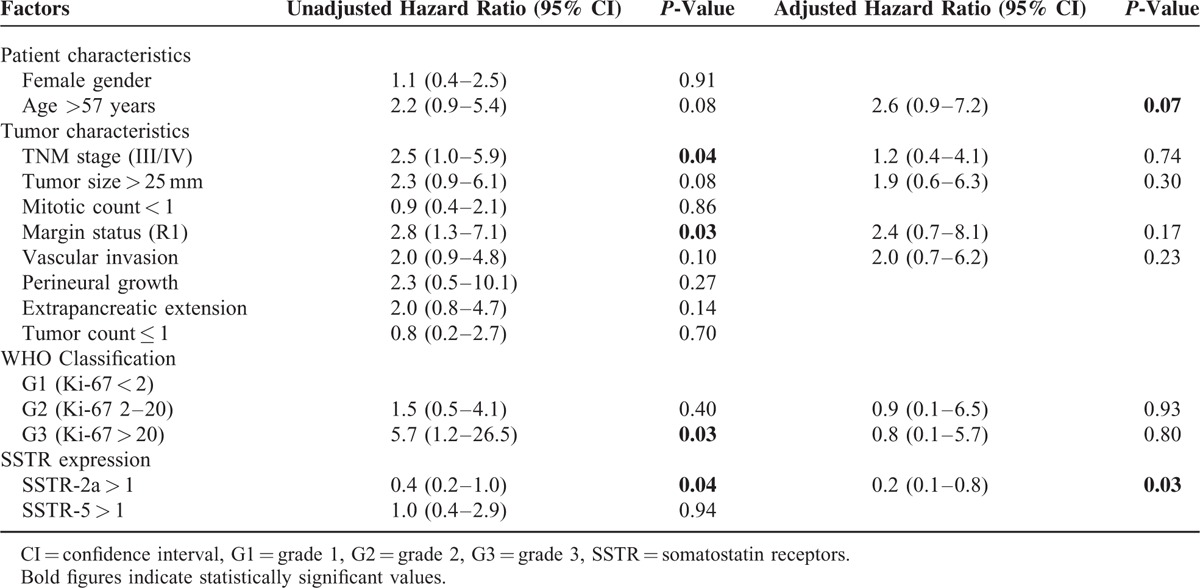
Univariate and Multivariate Analyses for Survival in PNETs

### Survival Analysis

**FIGURE 1:**
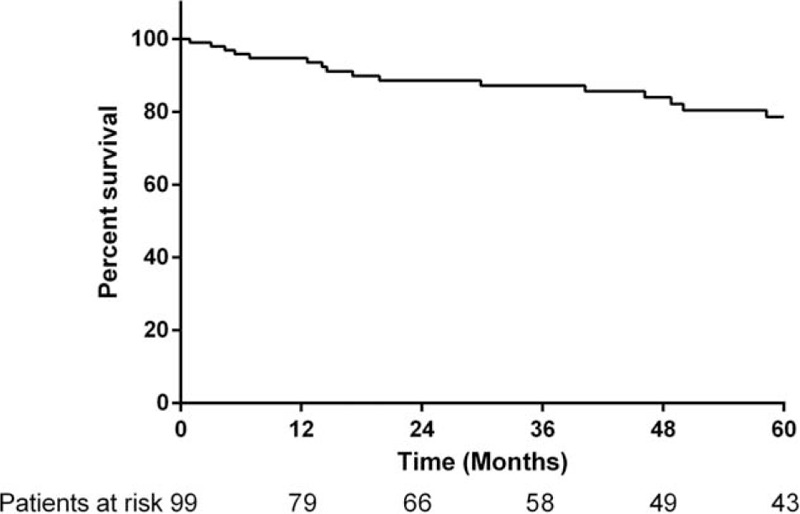
Kaplan–Meier analysis showing overall survival PNET patients (n = 99).

**FIGURE 2:**
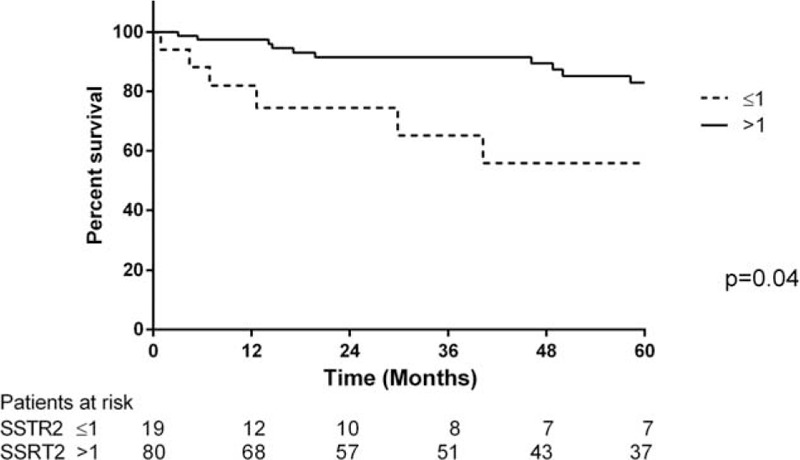
Comparison of overall survival in patients with PNET with SSTR-2a score ≤1 and SSTR-2a score >1.

**FIGURE 3:**
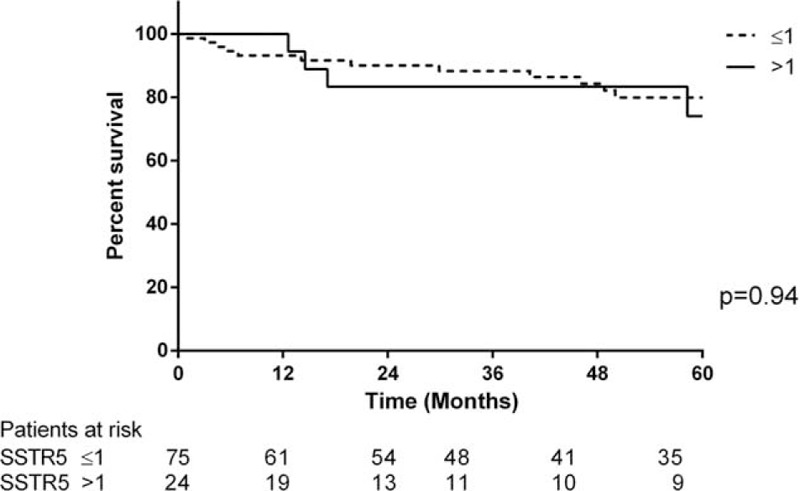
Comparison of overall survival in patients with PNET with SSTR-5 score ≤1 and >1.

**FIGURE 4:**
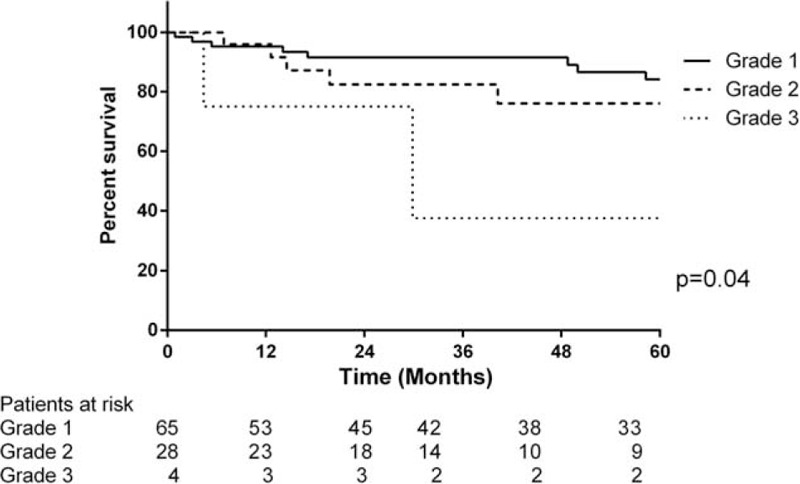
Comparison of overall survival in patients with PNET with grade 1, grade 2, and grade 3 PNETs based on their Ki-67 labeling index results.

The median follow-up for this cohort was 49 months (range 0.9–275.2 months). Twenty-two patients died during follow-up and 3 patients were lost to follow-up. The 30-day mortality rate was 0%. The overall cumulative survival rate was 94.8% at 1 year, 87.2% at 3 years, and 78.6% at 5 years (Figure 1). Survival rates in patients with an SSTR-2a score >1 was 97.5% at 1 year, 91.5% at 3 years, and 82.9% at 5 years, compared to those with an SSTR-2a score ≤1 having survival rates of 81.9% at 1 year, 65.1% at 3 years, and 55.8% at 5 years (log rank *P* = 0.04) (Figure 2). Among the patients with an SSTR-5 score >1, the survival rates were 100% at 1 year, 83.3% at 3 years, and 74.1% at 5 years. An SSTR-5 score ≤1 corresponded with survival rates of 93.2% at 1 year, 88.3% at 3 years, and 79.9% at 5 years (log rank *P* = 0.94) (Figure 3). Seventeen patients died due to PNETs, which resulted in a disease-specific cumulative survival of 82.8% during follow-up. The median disease specific survival was 29 months. Kaplan–Meier analysis showed that an SSTR-2a score of ≤1 was significantly associated with disease-specific survival (*P* = 0.02) (Figure 4).

In the univariate analyses, TNM stage III or IV (HR: 2.0, 95% CI: 1.0–5.9), Ki-67 labeling index >20 (HR: 5.7 and 95% CI: 1.2–26.5), and R1 status (HR: 1.08, 95% CI: 1.1–7.0) were associated with worse survival. A SSTR-2a score >1 (HR: 0.4 and 95% CI: 0.2–1.0) corresponded with better survival rates. In multivariate analyses, SSTR-2a >1 (HR: 0.2 and 95% CI: 0.1–0.8) was an independent positive prognostic indicator. A high-grade tumor as per the WHO classification was not found to be an independent prognostic indicator (HR: 0.8 and 95% CI: 0.1–5.7).

## DISCUSSION

In this present study of 99 surgically resected PNETs, tumor characteristics, histopathology, and patient outcomes were studied to ultimately determine the significance of SSTR-2a and SSTR-5 as prognostic markers for survival. In univariate analyses, it was found that tumor stage III/IV and Ki-67 labeling index >20% (ie, WHO grade 3) and R1 status were negative prognostic indicators, while the presence of SSTR-2a (score >1) was associated with improved survival. Multivariate analyses revealed that SSTR-2a score >1 was an independent positive prognostic indicator, while Ki-67 index and tumor stage were not found to be independent markers of prognosis.

Okuwaki et al published their study in 2013 that included 79 patients with PNETs. They reported the impact of SSTR-2a on survival outcomes in PNETs, correlating the presence of SSTR-2a with clinicopathological features, and concluded that SSTR-2a score of 0 is an independent negative prognostic factor for survival (HR: 3.6, 95% CI: 1.3–9.7). Their study exclusively focused on SSTR-2a, which is only one of the clinically relevant somatostatin receptors.^15^ This was preceded by a study in 2009 by Corleto et al, which focused on SSTR-2a and SSTR-5 expression in 33 cases of neuroendocrine tumors, not exclusive to PNETs. They demonstrated that cases with low SSTR-2a combined with low SSTR-5 expressions and Ki-67 ≥2 had poor survival outcomes in neuroendocrine tumors. Limitations to their study included the lack of a multivariate analysis to adjust for confounding factors, the limited number of cases included in the study and the generalized locations of the neuroendocrine tumors.^16^ To our knowledge, this present study is the largest study to date that investigates the impact of SSTR-2a on survival outcomes in a large number of PNETs, and the first study that combines the clinicopathological features and impact of SSTR-5 in an adjusted regression analysis.

Somatostatin is an endogenous peptide hormone that inhibits cellular proliferation and the secretion of certain endocrine hormones.^17,18^ Based on this principle, somatostatin analogs, such as octreotide and lanreotide, have been used for many years for symptomatic management of functional neuroendocrine tumors.^9^ The impact that somatostatin analogs have on neuroendocrine tumors relies on the expression of a range of somatostatin receptors (1–5), to which they have the highest affinity to SSTR-2a and a high affinity to SSTR-5.^3,7,9^ There is evidence to suggest that SSTR-2a and SSTR-5 expressions also influence and downregulate pancreatic carcinogenesis, angiogenesis, and initiate apoptosis.^17,19,20^

Consistent with Okuwaki et al^15^, this study showed that having a lower expression of SSTR-2a correlates with reduced overall survival, while a higher SSTR-2a expression (>1) corresponds with significantly improved survival rates. This study also showed that SSTR-2a is more widely expressed in well-differentiated (grade 1) neuroendocrine tumors compared to poorly differentiated tumors, supporting current literature that concludes neuroendocrine tumors have sparse SSTR-2a expression in higher grades (Table 2).^19,21^ This finding can be extrapolated to suggest that patients with poorly differentiated tumors have limited noninvasive treatment options secondary to the lack of SSTR-2a receptors and SSTR-2a expression is depleted due to the poor differentiation of tumors.

While 75% of cases (n = 18) with SSTR-5 expression were low-grade tumors, the majority of cases in this study did not express SSTR-5, and SSTR-5 expression did not significantly correlate with survival outcomes. There is evidence to suggest that SSTR-5 is expressed in neuroendocrine tumors and has an antiproliferative role.^19,22^ However, this did not translate to improved survival outcomes in cases with SSTR-5 score >1 in the present analysis of PNETs. This could be due to the majority of cases having an SSTR-5 score of 0 (64.6%). Analysis of the survival outcomes in cases with SSTR-5 score ≤1 and >1 might be subject to type I error.

Grade 3 PNETs were significantly associated with a poorer outcome in univariate analysis, but grading was not an independent prognostic indicator for survival after adding SSTR-2a to the model, which is in contrast to previous literature findings.^15,23^ One reason for this could be that our sample size of grade 3 tumors was very small, with only 4 cases in this group, compared to a relatively even distribution among the other grades.

The present study has several limitations. A retrospective assessment was performed on a cohort treated over 20 years. During this period, surgical resection and systemic treatment have advanced considerably, which may have altered patient outcomes overtime.^5^

Neuroendocrine tumors may demonstrate significant heterogeneity in their Ki-67 proliferative index. Currently based on the ENET/WHO guidelines the assessment of Ki-67 is based on the areas of tumor demonstrating the highest Ki-67 rates in whole stained sections.^11^ However in this study we based the Ki-67 proliferative index on the expression of Ki-67 in two 1 mm diameter cores in a TMA. While we recognize the potential for sampling artifact to underestimate the Ki-67 index when performed on TMA rather than whole sections, 42 (42%) of our cases also underwent Ki-67 proliferative index assessment on whole sections at the time of diagnosis and in our study we found a very significant correlation (*P* = 0.02) between the Ki-67 proliferative index as assessed on TMA with that found on whole sections in these cases. Therefore, despite this potential limitation to the study, the Ki-67 proliferation index based on the current TMA assessment remains a useful marker which strongly correlates with Ki-67 assessment on whole sections.

There were also imbalances in the distribution of cases across the grades of PNETs, with a much larger number of grade 1 PNETs compared to grades 2 and 3. Additionally as recommended by WHO 2010 guidelines,^10^ grading of cases with discrepant mitotic rates and Ki-67 indices was based on the highest grade which was invariably the Ki-67 proliferation index. While there is strong evidence for this approach in low-grade (grade 1 as compared to grade 2 tumors), it has recently been suggested that tumors with mitotic rates in the grade 2 range but Ki-67 in the low grade 3 range are significantly less aggressive than tumors with concordant grade 3 mitotic count and proliferative indices.^24,25^

An ideal study would be prospective, within a short, constrained time frame, with equal number of cases with grade 1, grade 2, and grade 3 tumors. This would assist in appropriately identifying the impact of SSTR-2a and SSTR-5 expression on survival outcomes within the grades of PNETs and comparing the survival rates of SSTR-2a expression in PNETs to Ki-67 labeling index. Nonetheless, given that PNETs are rare tumors, this study remains the largest to date of its kind.

In conclusion, the present study identified SSTR-2a expression as an independent prognostic factor for survival. Multivariate analysis showed that SSTR-2a expression is a stronger predictor for survival compared to Ki-67 labeling index, which is currently the marker for grading PNETs. Validation within a prospective patient cohort or if possible, within recently published or ongoing randomized trial populations is warranted. Reassessment of the current criteria for the classification of PNETs based on Ki-67 may be warranted in the future, and there may be a need to broaden the classification to incorporate biochemical markers of prognosis including SSTR expression. Improved insight into the biochemical mechanisms that are associated with PNET progression is necessary to derive effective curative treatments, given that currently, surgical resection still remains the only chance of cure.
